# An Integrated Artificial Intelligence Framework for Nanomedicine Engineering

**DOI:** 10.1002/advs.78125

**Published:** 2026-10-26

**Authors:** Meichen Dong, Qin Luo, Xinyi Ding, Chaoqi Lu, Ru Zhang, Qingqing Tan, Zhiqing Pang, Defang Ouyang, Dongsheng Li, Jiaxuan Xia, Jianxin Wang

**Affiliations:** ^1^ School of Pharmaceutical Sciences, Fudan University & amp, Key Laboratory of Smart Drug Delivery, Ministry of Education State Key Laboratory of Advanced Drug Formulations for Overcoming Delivery Barriers Shanghai China; ^2^ State Key Laboratory of Quality Research in Chinese Medicine, Institute of Chinese Medical Sciences (ICMS) University of Macau Macau China; ^3^ Microsoft Research Asia Shanghai China; ^4^ Quzhou Fudan Institute Quzhou Zhejiang,China

**Keywords:** artificial intelligence, closed‐loop optimization, data harmonization, high‐throughput validation

## Abstract

The translation of nanomedicines is frequently bottlenecked by trial‐and‐error optimization. Integrating artificial intelligence (AI) transforms this process into a closed‐loop, experimentally grounded discovery ecosystem. This Review provides a roadmap for this paradigm, demonstrating how AI, high‐throughput testing, and nanoscale design converge to generate clinically translatable nanotherapeutics. We emphasize the necessity of standardized multimodal data—spanning pharmacokinetic, biodistribution, and drug‐release profiles—anchored strictly to validated biological phenomena. Furthermore, we evaluate an algorithmic spectrum, ranging from classical machine learning to physics‐informed neural networks (PINNs) and reinforcement learning (RL), to align models with specific formulation objectives. To bridge computational design and empirical reality, we examine how high‐throughput technologies, such as robotic laboratories and in vivo DNA‐barcoding multiplexing, operationalize the “Design‐Make‐Test‐Analyze” (DMTA) cycle for dynamic model recalibration. Ultimately, this review demonstrates how integrating AI, physical constraints, and automated validation accelerates the rational design of next‐generation nanomedicines.

## Graphical abstract

This review outlines a closed‐loop AI ecosystem for nanomedicine engineering. By synergizing multimodal data harmonization, objective‐driven AI architectures, and automated high‐throughput validation, this “Design‐Make‐Test‐Analyze” (DMTA) workflow transforms traditional heuristic formulation into a predictive, self‐optimizing paradigm, accelerating the translation of next‐generation nanotherapeutics.

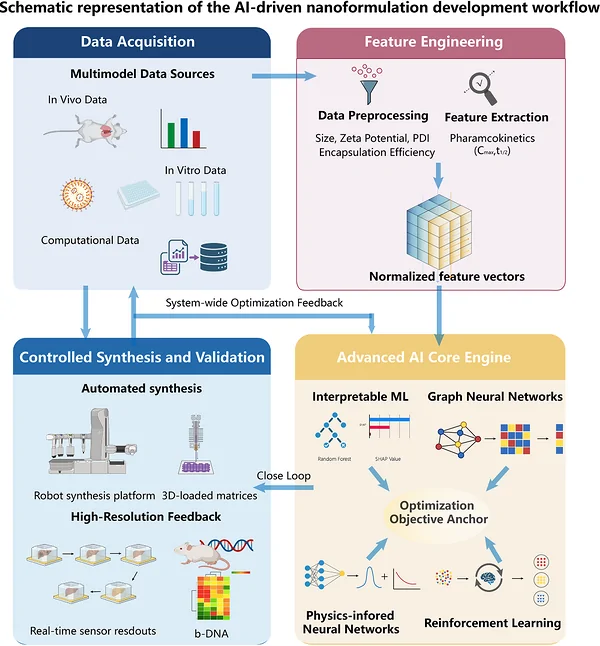

**Abbreviations**
%ID/g: Percentage of Injected Dose per Gram
3R: Replacement, Reduction, and Refinement
A3C: Asynchronous Advantage Actor‐Critic
ABM: Agent‐Based Model
ADME: Absorption, Distribution, Metabolism, and Excretion
AI: Artificial Intelligence
ANN: Artificial Neural Network
AUC: Area Under the Curve
bDNA: Branched DNA
CNN: Convolutional Neural Network
Cp: lasma Concentration
DL: Deep Learning
DMTA: Design‐Make‐Test‐Analyze
DNN: Deep Neural Network
DQN: Deep Q‐Network
FAIR: Findable Accessible Interoperable and Reusable
FDA: Food and Drug Administration
GAN: Generative Adversarial Network
GCN: Graph Convolutional Network
GNN: Graph Neural Network
GPR: Gaussian Process Regression
HDL: High‐Density Lipoprotein
HLB: Hydrophilic‐Lipophilic Balance
HPLC: High‐Performance Liquid Chromatography
ICMS: Institute of Chinese Medical Sciences
ICP‐MS: Inductively Coupled Plasma Mass Spectrometry
IVIVC: In Vitro‐In Vivo Correlation
IVIVE: In Vitro‐In Vivo Extrapolation
IVR: In Vitro Release
KNN: k‐Nearest Neighbors
LASSO: Least Absolute Shrinkage and Selection Operator
LightGBM: Light Gradient Boosting Machine
LIME: Local Interpretable Model‐agnostic Explanations
LNP: Lipid Nanoparticle
logP: Octanol‐Water Partition Coefficient
LSTM: Long Short‐Term Memory
MD: Molecular Dynamics
MIDD: Model‐Informed Drug Development
ML: Machine Learning
MRI: Magnetic Resonance Imaging
PBPK: Physiologically Based Pharmacokinetic
PCA: Principal Component Analysis
PD: Pharmacodynamic
PDI: Polydispersity Index
PEG: Polyethylene Glycol
PET: Positron Emission Tomography
PINN: Physics‐Informed Neural Network
PK: Pharmacokinetic
PK/PD: Pharmacokinetic/Pharmacodynamic
pKa: Acid Dissociation Constant
PPO: Proximal Policy Optimization
R2: Coefficient of Determination
RF: Random Forest
RFE: Recursive Feature Elimination
RL: Reinforcement Learning
RNN: Recurrent Neural Network
SHAP: SHapley Additive exPlanations
SPECT: Single‐Photon Emission Computed Tomography
SQL: Structured Query Language
SVM: Support Vector Machine
SVR: Support Vector Regression
t_1/2_
: Half‐life
TCN: Temporal Convolutional Network
Tg: Glass Transition Temperature
Tox: Toxicity
XAI: eXplainable Artificial Intelligence
XGBoost: eXtreme Gradient Boosting

Nanomedicines have exhibited significant potential for drug delivery within pharmaceutical development, circumventing the major barrier of targeted tissue accumulation. By resolving intrinsic limitations regarding solubility, circulating half‐life, and biodistribution, these engineered systems substantially enhance overall druggability—thereby substantially optimizing in vivo pharmacokinetic and pharmacodynamic (PK/PD) profiles [1, 2]. Validating this therapeutic paradigm, various formulations—including liposomes, polymeric micelles, and lipid nanoparticles (LNPs)—have successfully transitioned into commercial markets. Specifically, lipid‐core and hybrid polymeric nanocarriers have been extensively documented to improve the solubility, stability, and biodistribution of therapeutics across diverse clinical indications, spanning oncology to infectious diseases [3, 4]. A definitive milestone in this trajectory was the 2020 authorization of SARS‐CoV‐2 vaccines; the utilization of LNPs to encapsulate antigenic mRNA substantiated the clinical utility of nanoscale delivery architectures. The success of this mRNA platform elucidates how lipid‐based encapsulation confers essential stability and protection, ultimately translating inherently fragile biomolecules into viable therapeutics capable of efficient intracellular delivery [5, 6].

Despite these triumphs, the overarching performance of nanotherapeutics is governed by a highly complex, interconnected matrix of physicochemical parameters—encompassing particle size, zeta potential, lipid composition, the pKa of ionizable lipids, release kinetics, and surface modifications—which collectively dictate the in vivo fate of the delivery system. The high‐dimensional and non‐linear relationships inherent in these variables render rational design via traditional empirical experimentation highly challenging. Consequently, the field faces historically low rates of successful clinical translation [7], underscoring an urgent need for automated platforms that generate reproducible and mechanistically interpretable design principles. Amidst the myriad physicochemical and biological parameters dictating formulation efficacy, a triad of interdependent factors—specifically, PK/PD, in vivo biodistribution, and controlled drug release—constitutes the core characterization profile of nanomedicines. Within this framework, PK/PD parameters govern systemic exposure alongside the resultant therapeutic response, whereas biodistribution delineates site‐specific accumulation and targeting efficiency. Concurrently, release kinetics dictate the spatiotemporal retention characteristics of the active pharmaceutical ingredient. Collectively, these critical determinants not only elucidate the mechanistic interactions of nanoparticles within multiple biological systems but also serve as the ultimate prerequisites for achieving clinical translatability and securing regulatory approval.

Artificial intelligence (AI) emerges as a powerful paradigm, enabling the precise optimization and predictive modeling of complex, multidimensional parameters. The integration of AI algorithms—ranging from machine learning to deep learning networks—has proven instrumental in decoding the intricate biological interactions of nanoparticles, fundamentally reshaping the trajectory of smart drug delivery and precision medicine [8, 9, 10]. By anchoring on the critical triad of PK/PD, in vivo biodistribution, and controlled release kinetics, researchers can establish a systematic, AI‐driven framework to rigorously evaluate, model, and optimize the overarching performance of nanotherapeutics. Computational models trained on extensive empirical datasets possess the capability to decipher optimal permutations of formulation variables—thereby engineering ideal PK/PD profiles, accurately forecasting spatiotemporal biodistribution, and simulating release kinetics under physiological conditions [11, 12]. However, isolated computational predictions remain inherently limited. To fundamentally transition formulation design from heuristic exploration into a programmable discipline, AI must be embedded within a closed‐loop, experimentally grounded discovery ecosystem. This paradigm merges virtual high‐throughput screening directly with automated nanoscale synthesis and multiplexed biological testing, ensuring that computational predictions are continuously validated and recalibrated.

To delineate this frontier, this Review provides a critical roadmap for building and operationalizing these closed‐loop discovery ecosystems (Figure 1). By structuring our discourse along an interconnected workflow—spanning rigorous multimodal data harmonization, objective‐driven algorithm selection, and high‐throughput empirical validation—we demonstrate how AI, robotics, and nanoscale design principles must synergistically converge. Ultimately, by highlighting state‐of‐the‐art case studies alongside persistent translational challenges, we map the trajectory for generating reproducible, mechanistically interpretable, and clinically translatable nanomedicine candidates [13, 14].

**FIGURE 1:**
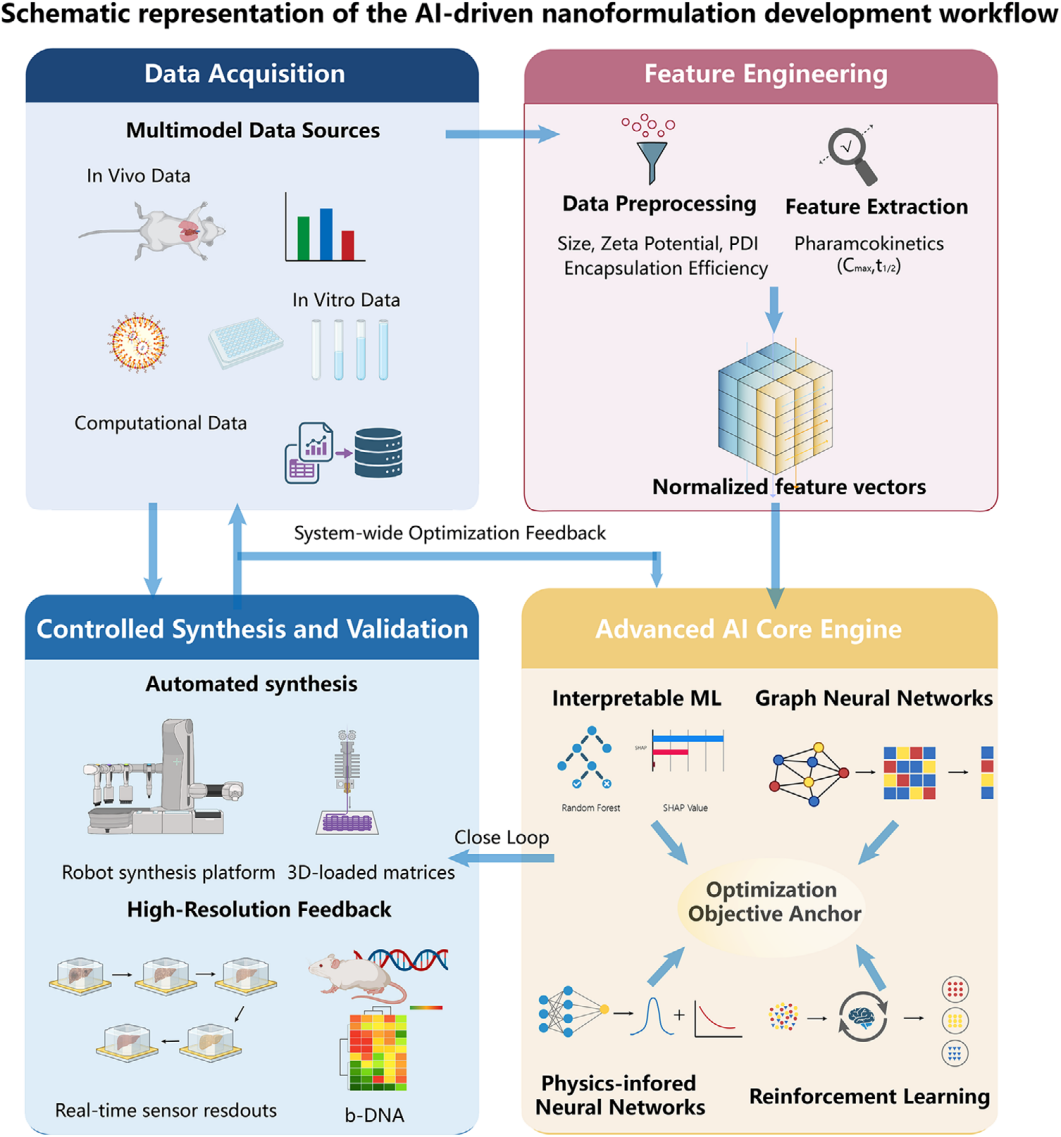
Schematic of the AI‐driven closed‐loop workflow for nanomedicine development. The ecosystem integrates multimodal data acquisition, feature engineering, objective‐driven AI modeling, and automated high‐throughput validation to iteratively optimize nanoformulations.

## 1 Data Acquisition

Data acquisition serves as the bedrock for any AI framework within nanomedicine design, as the predictive fidelity and generalizability of AI models are intrinsically contingent upon the scope, quality, and heterogeneity of the constituent training datasets. In the context of nanotherapeutic development, data must holistically encapsulate physicochemical, pharmacokinetic, pharmacodynamic, targeting, and drug‐release profiles—parameters that collectively govern the biological fate of nanoparticles. These data are typically bifurcated into experimental or computational streams across three primary functional categories: PK/PD behavior, targeting and biodistribution, and drug release kinetics. Each category necessitates distinct methodological approaches and data modalities; accordingly, this section delineates the diverse data acquisition strategies utilized to inform these frameworks.

### 1.1 Data Acquisition for PK/PD Modeling

PK/PD datasets characterize the longitudinal behavior of nanomedicines within biological systems, specifically elucidating how drug exposure translates into pharmacological efficacy. In vivo pharmacokinetic investigations yield critical quantitative metrics—such as plasma concentration‐time curves, area under the curve (AUC), half‐life (t_1/2_), maximum concentration (C_max_), and tissue distribution ratios. These parameters, traditionally derived from mass spectrometry, fluorescence imaging, or inductively coupled plasma mass spectrometry (ICP‐MS), constitute the empirical foundation for pharmacokinetic profiling. Such datasets are indispensable for correlating nanoparticle architecture with therapeutic outcomes and enabling AI‐driven predictions of systemic absorption, distribution, metabolism, and excretion (ADME).

Complementing these are In Vitro‐In Vivo Correlation (IVIVC) datasets, which provide mechanistic insights into the correspondence between in vitro dissolution, degradation, or release rates and subsequent in vivo plasma exposure. Often acquired via microdialysis or perfusion‐based assays, these datasets have been significantly augmented by AI‐based hybrid regression models, which enhance the correlation between IVIVC metrics and pharmacokinetic parameters, thereby refining early‐stage exposure forecasting and dosage optimization [15, 16].

Furthermore, computational and simulation‐derived datasets—including those generated from physiologically based pharmacokinetic (PBPK) or molecular dynamics (MD) simulations—augment empirical findings by predicting tissue permeability, clearance, and organ accumulation under diverse physiological constraints. Recent advancements have seen the development of AI‐assisted PBPK frameworks capable of inferring PK/PD outcomes directly from physicochemical descriptors—such as particle size, zeta potential, and lipid composition—demonstrating high concordance with experimental observations [17, 18, 19]. Integrating AI with PK/PD modeling not only enables the accurate simulation of in vivo nanoparticle fate and tissue‐specific sequestration patterns but also effectively bridges the chasm between experimental observations and clinical reality [7, 15, 20], thereby accelerating the bench‐to‐bedside transition.

In addition to primary research data, curated public repositories—such as ADMETlab, ChEMBL, and DrugBank—offer an extensive array of PK/PD descriptors (e.g., octanol‐water partition coefficient logP, solubility, and permeability coefficients). These standardized features serve as critical inputs for AI training and parametric prediction within nanomedicine models [21, 22]. To systematically categorize these fragmented resources, Table 1 aggregates the core multimodal databases currently driving AI nanomedicine development. As delineated in the table, distinct AI applications necessitate specific data modalities; for instance, chemical informatics platforms like ChEMBL and DrugBank provide the standardized logP and solubility descriptors critical for forecasting systemic drug exposure.

**TABLE 1:** Databases for AI Development of Nanomedicines.

Database / Platform	Primary Data Modality	Key Extracted Features	Primary AI Application in Nanomedicine
ADMETlab [23]	Pharmacokinetics & Toxicity	PK/PD descriptors such as octanol‐water partition coefficient (logP), solubility, and permeability coefficients.	Serves as critical inputs for AI training and parametric prediction of drug exposure.
ChEMBL [24]	Chemical Informatics	Extensive arrays of standardized PK/PD descriptors.	Provides essential parametric data to train AI models for predictive nanomedicine frameworks.
DrugBank [25]	Clinical & Pharmacological	Comprehensive PK/PD descriptors, including solubility and permeability characteristics.	Functions as structured input data for AI‐driven parametric forecasting and formulation modeling.
caNanoLab [26]	Nanomaterials & Bio‐interface	Protein corona “fingerprints” and surface ligand densities.	Provides structured inputs necessary for AI‐based in vivo biodistribution forecasting.
NanoCommons [27]	Nanoinformatics	Consolidated protein corona signatures and surface ligand density metrics.	Acts as a fundamental data source for AI models to predict targeting efficiency and spatial accumulation.
PolyInfo [28]	Polymer Materials	Polymer properties, including glass transition temperature (Tg), solubility, and cross‐linking density.	Enables the retrieval of specialized physical parameters to train AI models for drug release kinetics.
NanoHub [29]	Computational Nanotechnology	Specialized material parameters such as Tg, solubility, and cross‐linking density.	Serves as an indispensable resource for embedding physical and molecular constraints into AI frameworks.
Ai2Tox [30]	Nanomaterial Characterization & Protein Corona Profiles	Quantitative protein corona composition, nanoparticle physicochemical properties (e.g., size, surface modifications, lipid core molar ratio), and analytical protocols.	Predicting protein corona formation, modeling complex nano‐bio interactions, and guiding AI predictions for in vivo biodistribution, nanotoxicity, and targeting efficacy.

Collectively, these multi‐source datasets empower supervised, hybrid, and reinforcement learning models to forecast PK/PD trajectories based on formulation inputs. Conventional machine learning architectures, including Random Forest (RF), Gradient Boosting, and Support Vector Regression (SVR), have been effectively deployed to map formulation traits (size, composition, surface charge) to systemic exposure and clearance rates [20, 31]. Concurrently, Deep Neural Networks (DNNs) integrated with PBPK models have achieved near‐clinical precision in predicting tumor delivery efficiency and concentration‐time profiles [17]. Moreover, the emergence of RL and PINNs has facilitated the simulation of adaptive dosing and dynamic tumor accumulation, enabling real‐time control and optimization of PK/PD behavior [15, 16]. Ultimately, these integrated PK/PD datasets and AI models provide the quantitative scaffolding necessary to decode how nanomedicines circulate, bypass biological barriers, and exert therapeutic effects.

### 1.2 Data Acquisition for In Vivo Targeting and Biodistribution

Targeting datasets characterize the capacity of nanoparticles to navigate systemic circulation and accumulate within specific tissues, organs, or cellular compartments. These data encapsulate the convoluted interplay between nanoparticle physicochemical properties, protein corona formation, biological barriers, and receptor‐mediated uptake mechanisms. Notably, recent investigations have underscored that the composition and evolution of the protein corona can modulate biodistribution and targeting efficiency more significantly than intrinsic parameters such as size or surface charge alone [32]. For instance, cholesterol‐mediated alterations in the protein corona have been demonstrated to shift nanoparticle sequestration toward the liver and brain, elucidating a metabolic regulation of targeting behavior [33]. To inform AI‐driven frameworks, several pivotal data sources and acquisition methodologies are delineated below.

At the organ level, investigations into in vivo biodistribution typically leverage Positron Emission Tomography (PET), Single‐Photon Emission Computed Tomography (SPECT), and Magnetic Resonance Imaging (MRI) to quantify nanoparticle accumulation. A salient example is the use of ^89^Zr‐labeled high‐density lipoprotein (HDL) nanoparticles, where PET imaging successfully visualized tumor uptake in patients with esophageal cancer, thereby validating their selective localization and clinical safety in humans [34]. At the cellular and subcellular echelons, uptake studies rely predominantly on confocal microscopy and flow cytometry. At this scale, the protein corona remains a decisive factor in dictating internalization pathways, as diverse serum‐derived coronae trigger distinct receptor‐mediated endocytic mechanisms [35]. Proteomic profiling has further revealed that specific proteins, such as apolipoproteins, can either enhance or inhibit uptake by particular cell types [36]. Consequently, datasets characterizing the protein corona and bio‐interface are paramount for forecasting biodistribution [37]. Comparative studies between PEGylated and non‐PEGylated nanoparticles indicate that surface chemistry profoundly influences corona stability and macrophage recognition [38], enabling the rational design of directed or artificial coronae to actively modulate targeting selectivity [39]. Computational modeling further complements empirical data by simulating receptor‐ligand interactions and corona dynamics, as highlighted by recent AI‐integrated frameworks for nano‐bio interaction prediction [40]. Conversely, bio‐interface repositories such as NanoCommons, caNanoLab and Ai2Tox (Table 1) supply the quantitative protein corona “fingerprints” necessary for modeling complex nano‐bio interactions and predicting in vivo targeting efficacy [41].

Machine Learning (ML) models trained on these integrated datasets can identify the critical determinants of targeting efficiency, including surface charge, ligand density, and hydrophobicity. Deep Learning (DL) architectures—particularly Convolutional Neural Networks (CNNs) and Transformers—have been deployed to extract high‐dimensional spatial features directly from imaging datasets, thereby refining the predictive accuracy of organ‐level localization [40]. Ensemble methods, such as RF and XGBoost, have demonstrated superior predictive fidelity compared to traditional empirical models when forecasting nanoparticle distribution profiles based on physicochemical descriptors and corona characteristics [42]. Furthermore, transfer learning strategies enable the extrapolation of biodistribution data from animal models to human physiological contexts, addressing interspecies disparities in vascular architecture and immune response [43]. Collectively, targeting datasets provide the structural and functional scaffolding for AI‐driven predictions of in vivo fate, effectively bridging the gap between nanoscale physicochemical design and organ‐level therapeutic selectivity. It should be noted that to ensure mechanistic interpretability within AI frameworks, feature extraction must prioritize well‐validated, direct receptor‐ligand interactions rather than relying on abstract, overly complex, or controversial biological rationales. For example, AI‐driven models can be trained to systematically optimize the surface density and spatial orientation of specific functional ligands—such as sugar moieties protruding from ginsenoside‐based liposomes—to maximize binding affinity with overexpressed transporters like GLUT1 on the tumor cell surface [44]. By grounding predictive algorithms strictly in these explicit physical binding events, the resulting nanotherapeutic designs remain theoretically robust and experimentally reproducible.

### 1.3 Data Acquisition for Drug Release and Pharmacokinetics

Controlled drug release is a fundamental determinant of nanotherapeutic performance, as it dictates the local drug concentration and the duration of therapeutic exposure, thereby governing both efficacy and safety profiles. Data acquisition in this domain encompasses both the empirical quantification of release mechanisms and high‐fidelity computational modeling [45]. The primary data sources and methodologies for establishing robust, AI‐ready datasets for drug release are outlined in Figure 2. As shown in the workflow, compiling high‐quality release data requires strict inclusion criteria—such as verifying conventional formulation compositions and confirming the link between the in vitro release (IVR) test and the exact formulation used [46]. Following a rigorous appraisal of plot quality to mitigate performance and detection biases, manual data extraction and plot digitization generate the structured SQL databases essential for training AI models. Once this foundational data is secured, specific in vitro release assays—including dialysis, ultrafiltration, and high‐performance liquid chromatography (HPLC) quantitative analysis—provide time‐resolved datasets that characterize cumulative release profiles under diverse environmental constraints, such as varying pH levels, temperatures, and enzymatic concentrations [47].

**FIGURE 2:**
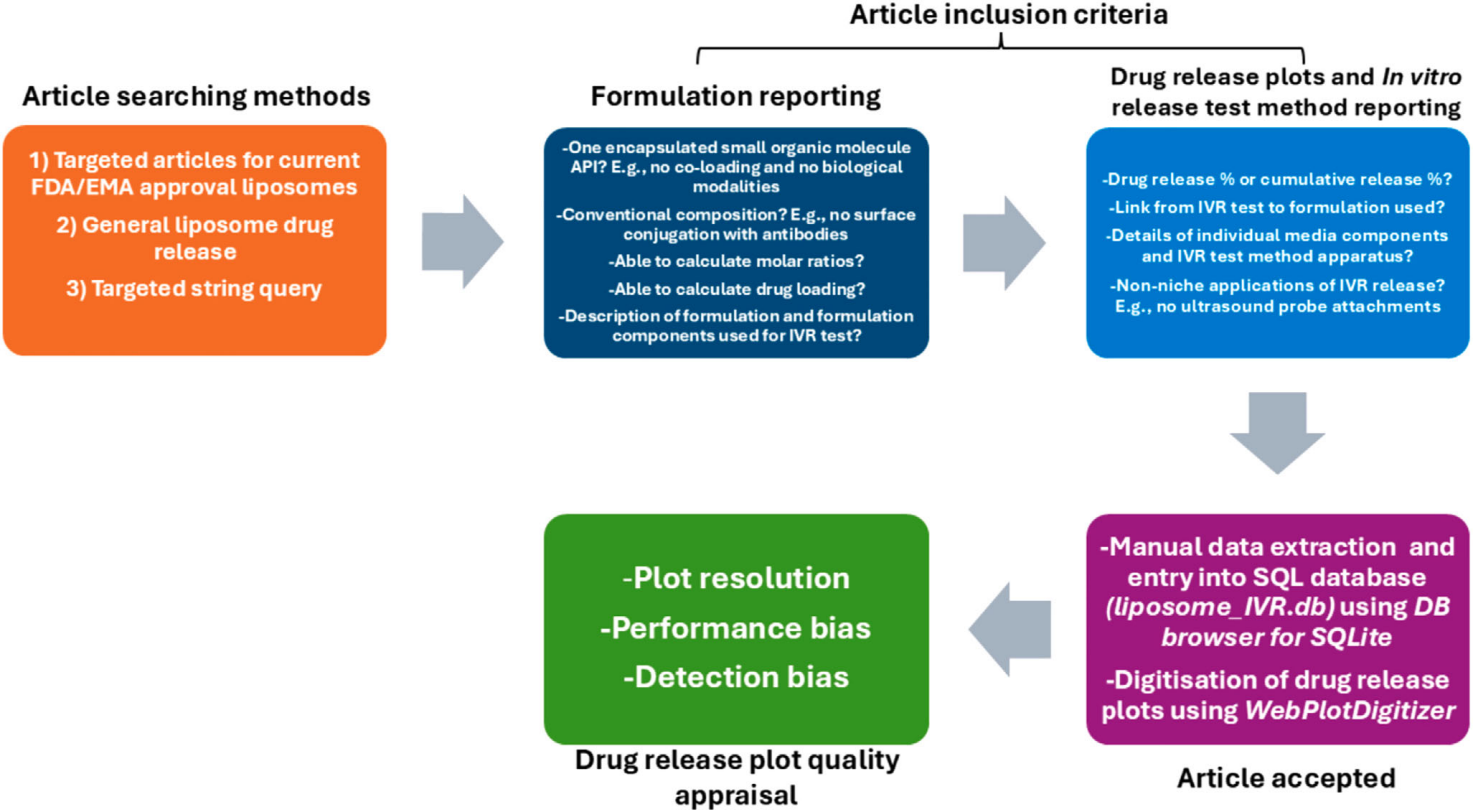
Workflow for establishing formulation parameters, in vitro release (IVR) test conditions, and a digital drug release database applicable for AI model training [46], licensed under CC BY 4.0.

The extraction of kinetic parameters (e.g., k_1_, k_2_, diffusion coefficients, and burst release fractions) typically utilizes classical mathematical constructs such as the Higuchi or Korsmeyer‐Peppas models. Notably, emerging Bayesian stochastic frameworks have significantly improved adaptive capabilities and uncertainty quantification [48]. These models, when integrated with microfluidic and high‐throughput screening systems, enable parallel formulation assessment, thereby generating robust training datasets for AI architectures [49]. Furthermore, computational datasets derived from MD and Monte Carlo simulations facilitate the characterization of diffusion and degradation at the molecular scale, serving as critical inputs for AI‐physics hybrid models [50]. Specialized polymer property repositories, such as PolyInfo and NanoHub (Table 1), remain indispensable for retrieving parameters including glass transition temperature (T_g_), solubility, and cross‐linking density [51].Supervised learning algorithms, including ANNs, RF, and Gradient Boosting, have been successfully deployed to model non‐linear release kinetics, consistently achieving coefficients of determination (R^2^) exceeding 0.9 [52]. Hybrid models, particularly PINNs that incorporate governing diffusion equations, have substantially enhanced the interpretability and generalizability of these predictions [50]. Concurrently, reinforcement learning and Generative Adversarial Networks (GANs) have enabled the adaptive control of formulation parameters—such as polymer ratios and solvent composition—to achieve target release trajectories [53].

In summary, data acquisition for AI‐guided nanomedicine design necessitates the multidimensional integration of PK/PD, targeting, and release datasets. Each category provides unique quantitative insights, spanning from systemic pharmacokinetics to intracellular targeting and molecular‐scale release mechanisms. The convergence of empirical data, computational simulations, and curated database‐derived information facilitates comprehensive model training, enabling the capture of the complete trajectory of nanomedicines in vivo. Looking forward, the development of unified, standardized, and FAIR‐compliant (Findable, Accessible, Interoperable, and Reusable) databases will be essential to further augment the predictive power and clinical translatability of AI‐driven nanotherapeutic design.

## 2 Data Cleaning and Feature Extraction

The predictive fidelity of AI models is fundamentally contingent upon the quality and representativeness of the input datasets. Raw data derived from disparate sources necessitate rigorous cleaning, normalization, and feature extraction to ensure consistency and reliability. Feature extraction serves as the critical pipeline for transforming raw variables—such as composition, pKa values, and lipid molar ratios—into high‐dimensional descriptors suitable for computational modeling. Principal Component Analysis (PCA), clustering, and unsupervised learning algorithms are frequently employed to identify dominant features and mitigate noise [54]; for instance, cluster‐based dimensionality reduction facilitates the elucidation of latent correlations between formulation parameters and performance metrics [55]. Ultimately, the reliability and performance of AI architectures are determined not only by the volume of available data but, more crucially, by the degree of data standardization and harmonization [56].Nanomedicine datasets originate from heterogeneous experimental and computational streams, each characterized by distinct protocols, instrumentation, and validation methodologies. Consequently, stringent data preprocessing and normalization are prerequisites for downstream analysis [57].

To overcome the inherent variability of data generated from disparate laboratories and experimental protocols, the adoption of FAIR (Findable, Accessible, Interoperable, and Reusable) principles is imperative. Effective harmonization relies on establishing standardized nanoparticle ontologies that unify nomenclature across diverse physicochemical and biological datasets [58]. Furthermore, the implementation of Automated Machine Learning (AutoML) preprocessing pipelines presents a robust solution for batch‐effect correction. By autonomously executing dynamic scaling, outlier filtration, and distribution alignment, these pipelines ensure that heterogenous datasets are systematically normalized into a unified, machine‐readable format, thereby safeguarding model reproducibility and generalizability across distinct experimental settings [59].

Standardized preprocessing workflows encompass error correction, scaling, imputation of missing values, and outlier detection through statistical and algorithmic filters [60]. Post‐cleaning, feature extraction converts empirical or physicochemical descriptors into representations optimized for AI modeling, thereby enabling robust predictions of in vivo fate. Advanced architectures, such as Graph Convolutional Networks (GCNs) and Transformers, further enhance model interpretability by deciphering the complex interdependencies among multifaceted features [61]. Recently, AI‐driven nanomedicine design platforms have integrated these methodologies to unify raw, noisy, and multiscale data into robust, machine‐readable structures [62], as illustrated in Figure 3. The process initiates with a highly heterogeneous data pool (Figure 3A), comprising sparse in vivo matrices, detailed in vitro cellular assays, and high‐density computational data. Because these raw matrices are often incomplete, an AI‐coordinated processing engine (Figure 3B) performs necessary data cleaning and imputation, utilizing known physical mechanisms to normalize the data without relying on overly restrictive mathematical assumptions. Finally, this processed data is embedded into a highly structured feature space (Figure 3C), yielding distinct vectors for physicochemical, pharmacokinetic, and bio‐distributional properties. This section summarizes the tailored data cleaning and feature extraction strategies categorized by their relevance to PK/PD, biodistribution, and drug release kinetics.

**FIGURE 3:**
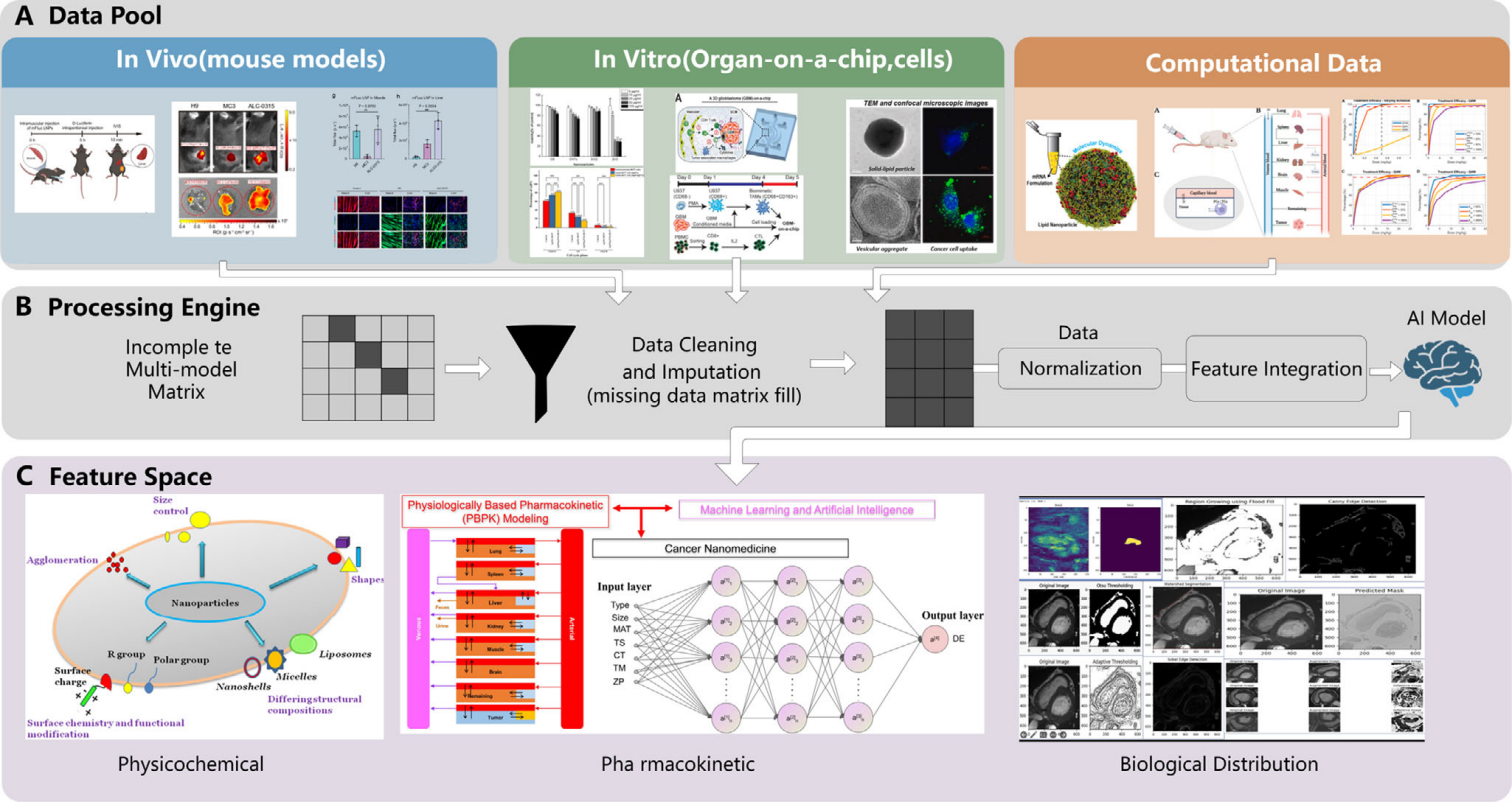
Schematic of multi‐source data integration and feature extraction for AI‐driven nanoparticle design. (A) Heterogeneous data pool comprising three sources with variable data density: sparse in vivo matrices (PK, biodistribution, toxicity) [83], detailed in vitro cellular assays (uptake, endosomal escape, cargo release, efficacy) [84, 85, 86], and high‐density computational data (molecular descriptors, binding energies, protein corona composition) [20, 87, 88]. (B) AI‐coordinated preprocessing pipeline performing data cleaning and matrix imputation to transform incomplete raw matrices into normalized, integration‐ready datasets. (C) The processed data are ultimately transformed into structured domain‐specific feature vectors encompassing three critical dimensions: physicochemical properties (hydrodynamic diameter, zeta potential, polydispersity index) [89], pharmacokinetic properties (maximum plasma concentration, elimination half‐life) [31], and biodistribution and biointerfacial properties (tissue accumulation, protein corona composition) [90].

### 2.1 Data Processing and Feature Extraction for PK/PD Modeling

PK/PD datasets typically consolidate time‐ and concentration‐dependent data derived from plasma and tissue samples. The cleaning and feature extraction of these data demand meticulous handling of dynamic, multiscale observations. Raw PK/PD datasets often contain heterogeneous time‐series measurements, such as plasma concentrations (C_p_), tissue accumulation, and excretion rates; these require normalization via spline interpolation, Z‐score transformation, or Kalman filtering to mitigate sampling inconsistencies [21]. Cross‐study integration utilizes body weight and surface area corrections to calibrate pharmacokinetic parameters—such as AUC, t_1/2_, and clearance rates—ensuring interspecies comparability [62]. AI models are trained on an extensive feature set encompassing experimental and physicochemical descriptors, including particle size, polydispersity index (PDI), zeta potential, lipid composition, pKa, and PEGylation density [31]. Biological context variables, such as plasma protein concentrations, physiological pH, and immune activation status, are likewise integrated to construct correlation matrices and non‐linear embeddings for accurate PK forecasting. Hybrid mechanistic models, which fuse PBPK structures with machine learning layers, impose physiological constraints that enhance stability in data‐scarce scenarios [15]. Despite significant progress, PK/PD datasets remain susceptible to experimental noise and interspecies scaling biases [17]. Comprehensive frameworks that synthesize Model‐Informed Drug Development (MIDD) with AI have demonstrated significant promise in reducing variability while preserving biological interpretability [63]. Correspondingly, computational nanotoxicology models exemplify how in vitro‐to‐in vivo extrapolation (IVIVE) can effectively harmonize datasets derived from disparate sources [64].

### 2.2 Data Cleaning and Feature Extraction for In Vivo Targeting and Biodistribution

Targeting‐related datasets encompass a broad spectrum of data ranging from macroscopic biodistribution to microscopic cellular localization, presenting substantial heterogeneity in dimensionality and format. Recent advancements in AI‐integrated imaging technologies have enabled three‐dimensional analysis of nanoparticle deposition and immune cell interactions with unprecedented spatial resolution [65]. Concurrently, multimodal imaging strategies—leveraging the synergy of MRI, PET, and fluorescence imaging—provide a complementary data foundation for mapping biodistribution, immune engagement, and pharmacokinetic trajectories [66].

Raw datasets typically include fluorescence intensity maps, radiotracer counts, and the percentage of injected dose per gram (%ID/g). Data cleaning procedures involve rigorous background subtraction, normalization against reference tissues (e.g., liver or blood), and logarithmic transformation to rectify signal skewness. Imaging datasets often necessitate pixel‐level denoising and registration, particularly in multimodal studies where geometric discrepancies exist between distinct imaging modalities [67]. At the cellular level, deep learning‐based segmentation methods—such as CNNs or U‐Net architectures—are deployed to automate the extraction of quantitative fluorescence and MRI features, thereby minimizing subjective human bias [68, 69]. Normalization against reference dyes and tissue controls further ensures cross‐experimental comparability, a critical requirement when integrating PET/SPECT or optical data. Robust data fusion strategies and registration workflows have been established to harmonize imaging intensities across diverse modalities [70]. Recent multimodal investigations have demonstrated that AI‐assisted image fusion and Monte Carlo simulation frameworks can synergistically optimize imaging resolution and biodistribution forecasting [71]. Such integrated models facilitate spatially resolved estimations of nanoparticle targeting efficiency across disparate tissue and cellular compartments.

Consequently, the extracted features optimized for AI modeling typically include: (i) Quantitative descriptors, such as fluorescence fold‐change, %ID/g, uptake constants (k_in_, k_out_), and signal‐to‐background ratios [68]; (ii) Structural descriptors, including particle size, aspect ratio, zeta potential, and surface ligand density [41]; and (iii) Bio‐interface descriptors, such as protein corona signatures, complement activation indices, and opsonization ratios—each exerting significant influence on biodistribution [40].However, targeting datasets remain inherently heterogeneous and susceptible to systematic bias. Fluorescence signals are frequently confounded by tissue scattering, while radiolabeling may inadvertently alter the physicochemical properties of nanoparticles, introducing artifacts into the resulting biodistribution profiles [72]. Furthermore, the lack of standardized ontologies for protein corona descriptors and ligand density measurements hinders cross‐study integration. Consequently, recent literature advocates for the implementation of explainable AI (XAI) frameworks—such as SHAP (SHapley Additive exPlanations) and LIME (Local Interpretable Model‐agnostic Explanations)—to quantify the contribution of each variable to model predictions [73]. These methodologies underscore the necessity of unified preprocessing, standardized feature ontologies, and XAI approaches for the robust modeling of nanotherapeutic targeting efficiency.

### 2.3 Data Cleaning and Feature Extraction for Drug Release Kinetics

Drug release datasets characterize the temporal evolution of therapeutic payloads from nanocarriers under defined physicochemical constraints. These data are pivotal for optimizing therapeutic efficacy and determining the minimum effective dose by establishing robust correlations between formulation design parameters and release kinetics [45]. The evolution of AI modeling has further enhanced the capacity to predict and modulate release dynamics across diverse polymeric, lipidic, and hybrid nanocarriers [49].Raw release data typically manifest as cumulative percentage‐release curves plotted against time, measured under varying pH, temperature, and enzymatic conditions.Prior to feature extraction, imperative preprocessing steps—such as noise reduction (e.g., Savitzky‐Golay filtering) and curve smoothing—are employed to ensure signal integrity [52]. To facilitate cross‐formulation comparability, datasets undergo temporal normalization (t/t_max_) and dose‐adjustment, while parameters for polymer and lipidic systems are standardized against encapsulation efficiency and surface area. This unified processing mitigates biases inherent in disparate experimental protocols, ensuring consistency in downstream modeling.

AI models leverage a synergy of kinetic and structural descriptors to capture release trajectories. For instance, experimental descriptors include cumulative release percentages at specific time intervals (e.g., 1, 4, 12, 24, and 48 h), initial burst release fractions, sustained‐phase rate constants, and residual drug content [48]. Material descriptors encompass polymer molecular weight, glass transition temperature (T_g_), hydrophilic‐lipophilic balance (HLB), lipid chain length, cross‐linking density, and porosity [50]. Furthermore, environmental descriptors—such as pH, ionic strength, and enzymatic activity—serve as critical covariates influencing degradation and diffusion mechanisms [74]. Deep and hybrid AI architectures, including ANNs, Graph Neural Networks (GNNs), and PINNs, demonstrate superior proficiency in modeling non‐linear release patterns. For example, GNNs can correlate T_g_ and pH‐sensitivity with sustained‐release coefficients, thereby enabling precise release profile forecasting without extensive empirical testing [52]. Furthermore, PINN frameworks that embed Fickian diffusion or degradation kinetics enhance model interpretability and robustness by constraining the learning process with governing physical laws [48, 50].

Upon completion of cleaning and standardization, all three data categories—PK/PD, targeting, and release—are transformed into numerical matrices or tensors compatible with AI model inputs. Continuous variables (e.g., size, zeta potential, fluorescence intensity) are normalized to zero‐mean, unit‐variance scales, while categorical variables (e.g., lipid composition, surface ligands) are subjected to one‐hot encoding [75]. Multimodal datasets, integrating imaging, kinetic, and physicochemical data, are consolidated via feature concatenation or multimodal Transformers capable of aligning latent feature spaces [61]. Recent applications of multimodal AI in clinical prediction tasks reveal that Transformer‐based fusion models outperform unimodal pipelines by embedding radiomics, kinetics, and biosignals into a unified vector space [76]. Feature extraction remains critical for optimizing interpretability and mitigating overfitting. Techniques such as Recursive Feature Elimination (RFE), LASSO regularization, and SHAP analysis are now widely deployed to rank the relative importance of high‐dimensional nanomedicine features [77]; the integration of LASSO and SHAP enhances model parsimony while bolstering transparency [78]. Within ensemble learning workflows, SHAP‐derived interpretability is increasingly coupled with meta‐classifiers to quantify the impact of specific physicochemical parameters—such as PEGylation density or protein corona composition—on predictive outcomes [79]. Moreover, XAI frameworks combining Shapley value decomposition with meta‐heuristic optimization are emerging as standard tools for deciphering “black‐box” neural networks [80]. These methodologies empower researchers to identify the most influential predictors (e.g., surface charge, diffusion rates, specific protein corona constituents) and visualize their non‐linear contributions to AI decision‐making. Ultimately, these curated datasets train supervised or hybrid AI models that map the nexus between nanomedicine design and in vivo fate. Multi‐view deep learning architectures, combining cross‐attention mechanisms and ensemble methods, have demonstrated robust generalization capabilities in pharmacokinetic prediction. AI‐driven optimization pipelines now facilitate the design of nanomedicines with bespoke particle size, surface chemistry, and release kinetics, transitioning experimental workflows into data‐efficient and interpretable platforms [49].

In summary, meticulous preprocessing, multimodal integration, and feature selection constitute the foundation for converting complex nanomedicine data into structured inputs suitable for AI processing. By standardizing PK/PD, targeting, and release descriptors—and quantifying key physicochemical drivers—researchers can construct transparent, generalizable, and high‐fidelity AI models that bridge the chasm between computational modeling and clinical translation [81]. Nevertheless, despite rapid progress, significant challenges persist: published datasets remain fragmented and heterogeneous due to inconsistent sampling conditions and temporal resolution. Furthermore, burst‐release artifacts induced by sampling errors or diffusion gradients introduce systematic bias [49]. Additionally, the limited scale of datasets concerning stimulus‐responsive systems (e.g., pH/enzyme‐triggered release) impedes model generalization [74]. Consequently, emerging hybrid strategies that synthesize mechanism‐based physical models with data‐driven AI hold significant promise for enhancing predictive reliability and advancing the paradigm of personalized nanomedicine design [82].

## 3 Model Selection and Training

The inherent variability in data modalities, sample sizes, and requirements for interpretability necessitates the selection of distinct AI architectures tailored to specific stages of nanomedicine development. Traditional ML models—including Support Vector Machines (SVM), k‐Nearest Neighbors (KNN), and RF—are widely deployed for small‐to‐medium‐scale datasets where computational efficiency and interpretability are paramount [56]. By leveraging physicochemical descriptors such as particle size, surface charge, and polymeric composition, these algorithms have demonstrated significant success in predicting nanoparticle stability, drug‐loading efficiency, and release parameters [91]. Conversely, DL architectures, such as CNNs and RNNs, excel at extracting non‐linear, hierarchical relationships from high‐dimensional datasets. Compared to conventional ML, these frameworks have achieved superior performance in forecasting pharmacokinetics, drug release kinetics, and cellular uptake patterns. In nanomedicine design, CNN‐based frameworks are frequently utilized to analyze microscopic and radiomic imagery for modeling targeting and biodistribution, whereas RNNs and Transformer‐based architectures are optimized for processing pharmacokinetic time‐series data [92].

Beyond conventional ML and DL, RL and PINNs extend predictive horizons by integrating optimization logic and physical priors. RL has been successfully implemented to identify optimal formulation parameters and adaptive dosing regimens in silico, effectively reducing the experimental search space [93]. Simultaneously, PINNs incorporate diffusion, reaction‐kinetics, and transport equations directly into the learning framework, enabling more physiologically grounded predictions of in vivo drug release and nanoparticle transport [57].

In practice, the selection of an AI model is predicated on specific objectives—whether predicting PK/PD profiles, targeting efficiency, or controlled‐release efficacy—as well as the nature of the data (scale, modality, and noise levels) and the requisite degree of interpretability. For instance, interpretable RF or SVM models are optimally suited for early‐stage screening, whereas hybrid DL or RL‐PINN architectures are better equipped for complex in vivo fate predictions that demand adaptive optimization [94], the strategic matching of model architectures to formulation objectives is conceptualized in the objective‐driven selection matrix in Figure 4. To navigate this algorithmic landscape, researchers must rely on distinct criteria for model selection based on data characteristics and specific formulation objectives. Classical ML architectures (e.g., RF, SVM) are the optimal choice for small‐to‐medium‐scale, highly structured datasets where model interpretability and feature importance ranking are paramount. Conversely, DL frameworks (e.g., CNNs, RNNs) are necessitated when processing high‐dimensional, unstructured data, such as spatial imaging or complex time‐series kinetics [95]. When predictive tasks require strict adherence to physiological or mechanistic boundaries, PINNs should be selected to constrain the learning process with governing physical equations [96]. Finally, for objectives centered on continuous, autonomous formulation refinement within a closed‐loop empirical environment, RL serves as the indispensable architecture [97]. As depicted, interpretable machine learning models (e.g., RF coupled with SHAP analysis) transparently guide early formulation screening. In contrast, complex structural characterizations require the latent space representations generated by Deep Learning and GNNs. To ensure theoretical consistency, PINNs enforce mechanistic fidelity using generalized physical constraints, while RL drives the continuous, autonomous adjustment of parameters. Collectively, these AI models are catalyzing the paradigm shift of nanotherapeutic design from heuristic, empirical exploration toward a predictive, data‐driven, and mechanism‐guided engineering process [98]. The following subsections illustrate the applicability of these models across diverse nanomedicine development scenarios.

**FIGURE 4:**
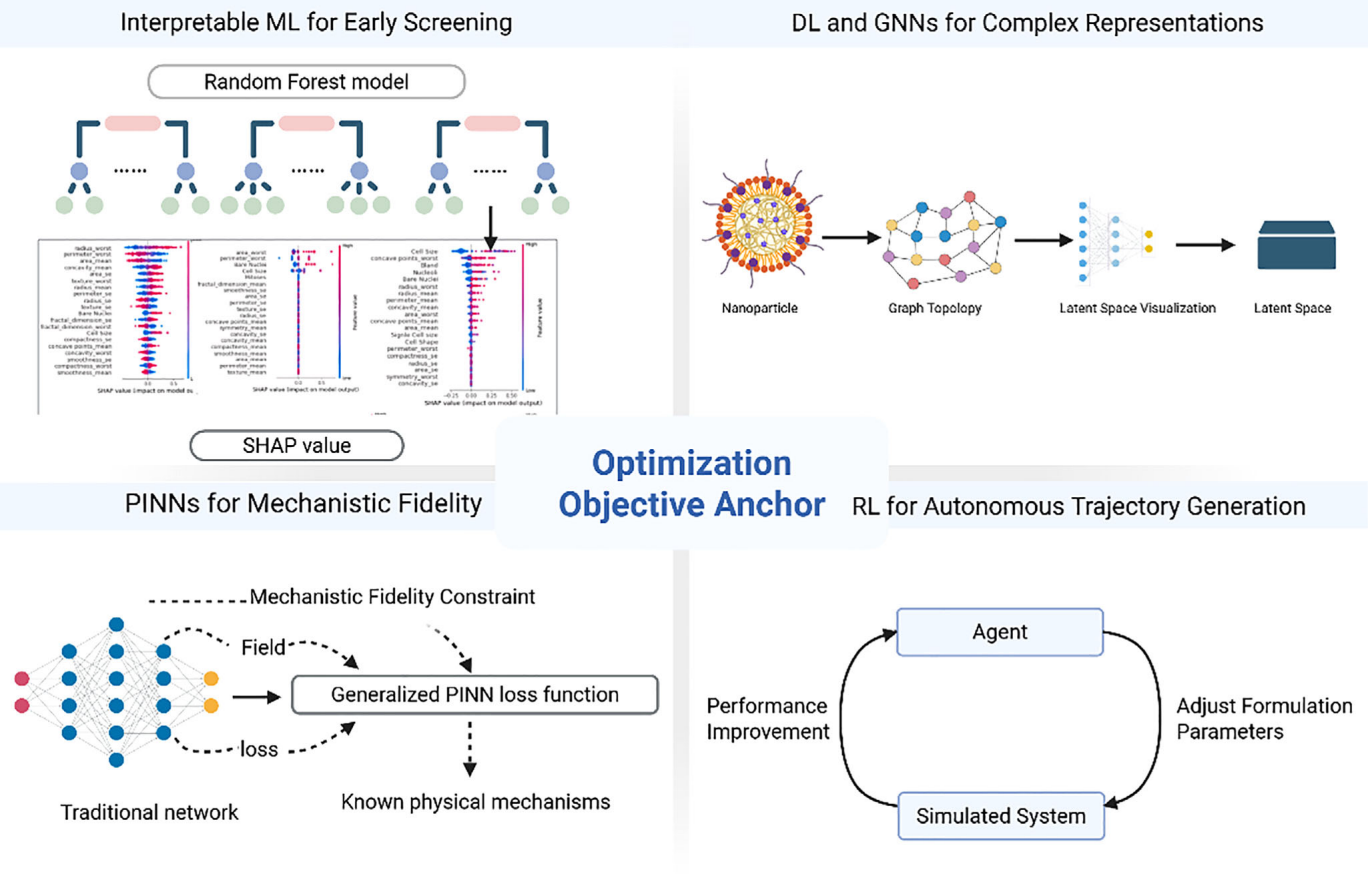
Objective‐Driven AI Algorithm Selection Matrix. A central optimization anchor unifies specialized AI architectures tailored to distinct stages of formulation development. Interpretable ML utilizes RF models and SHAP analysis to decode feature importance and transparently guide early screening [78]. For complex structural characterization, Deep Learning and GNNs map nanoparticle interactions into graph topologies, extracting high‐dimensional latent space representations. Bridging empirical data with theoretical rules, PINNs integrate known physical mechanisms into generalized loss functions to enforce mechanistic fidelity during training. Driving dynamic formulation design, Reinforcement Learning establishes an autonomous trajectory loop where an agent iteratively adjusts formulation parameters within simulated systems to maximize performance outcomes. (Created in https://BioRender.com).

### 3.1 Machine Learning Models

Traditional ML algorithms—such as RF, SVM, and Gradient Boosting Machines (XGBoost, LightGBM)—remain among the most robust models for processing structured, medium‐scale datasets, offering a precise balance between predictive accuracy and interpretability [99]. Among these, gradient‐boosting variants such as XGBoost and LightGBM facilitate rapid convergence and high predictive stability while maintaining transparency via feature importance analysis [100]. ML has been extensively utilized to model pharmacokinetic parameters—including AUC, C_max_, and clearance—by exploiting physicochemical features such as size, zeta potential, and lipid molar ratios. For example, RF and XGBoost have been successfully deployed in pharmacological contexts to forecast drug exposure and optimize dosing regimens, consistently outperforming classical regression models [101].

These ensemble models effectively capture non‐linear dependencies between formulation variables and pharmacokinetic responses, rendering them invaluable in the nanomedicine design workflow. Particularly when coupled with feature selection algorithms like SHAP or LASSO, traditional ML models can identify the most critical determinants of targeting efficiency, such as surface charge, ligand density, and protein corona composition. In relevant biomedical modeling, LightGBM and RF classifiers have demonstrated exceptional feature‐ranking capabilities in lipidomics and molecular targeting prediction, providing interpretable correlations between formulation traits and organ‐level biodistribution results. Furthermore, gradient boosting and Gaussian Process Regression (GPR) have proven effective in modeling non‐linear drug release kinetics and dissolution profiles, especially under fluctuating pH or enzymatic conditions. Boosting methods exhibit high computational efficiency in high‐dimensional feature spaces, achieving training speeds 10–1000 times faster than conventional RF [100]. Modern ML workflows increasingly incorporate cross‐validation, grid or Bayesian hyperparameter optimization, and Recursive Feature Elimination (RFE) to achieve an equilibrium between predictive performance and model transparency. For instance, recent optimization workflows employing RF, SVM, and Extreme Gradient Boosting (XGBoost) on biomedical datasets have demonstrated highly robust predictive performance and generalization capabilities while preserving model interpretability [102].

### 3.2 Deep Learning Models for Complex Feature Representation and Targeting Prediction

As datasets escalate in volume, dimensionality, and multimodality—integrating imaging modalities, molecular descriptors, and kinetic profiles—DL architectures emerge as the requisite tools for robust feature representation. Prevalent architectures, including ANNs, CNNs, RNNs, and Transformers, possess the capability to distill non‐linear, hierarchical relationships from complex datasets, thereby surpassing conventional machine learning models in both scalability and abstraction [61].

Regarding PK/PD modeling, recurrent architectures—such as RNNs and Long Short‐Term Memory (LSTM) networks—are exceptionally adept at capturing temporal dependencies within concentration‐time datasets, facilitating the extrapolation of plasma concentration curves or drug accumulation trajectories from early‐stage observations. These models have demonstrated high predictive fidelity in sequence‐based pharmacokinetic forecasting and molecular transport modeling [103]. In the domain of targeting and biodistribution, CNNs are extensively deployed to process imaging‐based datasets—including fluorescence, PET, and MRI—to extract spatial accumulation patterns of nanoparticles. Furthermore, by integrating CNNs with autoencoders or graph‐based encoders, researchers can derive the correlations between morphology and surface properties directly from microscopic imagery, thereby deepening the quantitative understanding of nanoparticle‐cell interactions [90]. In the context of release forecasting, fully connected ANNs or Temporal Convolutional Networks (TCNs) have been implemented to model the non‐linear nexus between formulation composition and cumulative release profiles. Such frameworks autonomously learn release kinetic parameters under heterogeneous environmental constraints (e.g., pH, enzymatic activity, temperature), enabling the precise prediction of sustained and burst release phases based on molecular and polymeric descriptors [104]. GNNs have further catalyzed advancements in this field by representing molecular and nanoparticle entities as graphs, facilitating the learning of topological‐dependent effects, such as lipid‐RNA packing and polymer cross‐linking density. While currently in the early stages of empirical validation within nanomedicine, preliminary studies corroborate that GNNs hold profound potential for characterizing three‐dimensional nanoparticle architectures and predicting structure‐function correlations within intricate delivery systems [105]. Practically, AI platforms such as AGILE and FormulationAI have successfully leveraged deep generative models to screen millions of candidate lipid structures and decipher composition‐performance mapping, significantly enhancing in vivo transfection efficiency [61]. The training of these DNNs necessitates large‐scale, high‐quality datasets and robust regularization strategies—including dropout, batch normalization, and weight decay—to mitigate overfitting. Furthermore, transfer learning and pre‐training on related materials or molecular datasets (e.g., protein structures or polymer databases) significantly augment model generalizability, particularly in scenarios where nanomedicine‐specific data remain constrained [106].

In summary, deep learning provides a flexible and potent framework for characterizing the non‐linear, high‐dimensional relationships underlying nanotherapeutic performance. Through the integration of hybrid architectures—including CNNs, RNNs, GNNs, and Transformers—AI systems are now capable of decoding complex, multiscale correlations across pharmacokinetics, targeting, and release domains, thereby paving the way for autonomous and adaptive nanomedicine design.

### 3.3 Reinforcement Learning (RL) and Closed‐Loop Optimization Frameworks

Reinforcement Learning (RL) represents a fundamental paradigm shift from passive predictive modeling to proactive, autonomous decision‐making. Unlike supervised models that merely fit existing datasets, RL agents learn through continuous environmental interaction—iteratively optimizing formulation parameters (state variables) to maximize predefined objectives (rewards), such as prolonged systemic circulation, enhanced targeting specificity, or precision‐controlled drug release. This transformation empowers AI systems not only to model nanotherapeutic performance but also to perform in silico autonomous formulation design and optimization.

In the realm of formulation discovery, RL agents iteratively adjust the ratios of lipids, surfactants, and excipients based on experimental or simulated feedback, effectively circumventing the need for exhaustive combinatorial screening. This methodology has been rigorously validated in molecular and formulation design, where deep reinforcement learning efficiently identifies optimal molecular architectures and excipient combinations [107]. It is critical to note that while these RL‐driven methodologies have achieved robust, quantitatively validated successes in broader molecular design [108] and continuous flow materials synthesis, their direct application in autonomous [109], multi‐component nanomedicine formulation is still emerging. Consequently, the projection of RL as a primary driver for fully autonomous nanotherapeutic discovery currently represents a conceptual extrapolation, drawing upon the proven precedents of chemical automation to define the next frontier of nanomedicine optimization. Within nanomedicine workflows, this approach significantly accelerates the optimization of lipid nanoparticles (LNPs) and polymeric carriers by implementing reward functions correlated with encapsulation efficiency, colloidal stability, or release uniformity.

When integrated into autonomous laboratories, RL agents serve as the decision‐making engine within closed‐loop experimental platforms, synergizing AI‐driven forecasting with real‐time synthesis and characterization. For instance, automated experimental systems utilizing safe reinforcement learning frameworks have been developed to optimize chemical and materials synthesis under conditions of high uncertainty [110]. Such robotic laboratories embody the philosophy of AI‐driven autonomous formulation, wherein RL agents continuously learn from feedback provided by robotic analysis—dynamically adjusting polymer ratios, solvent compositions, or thermal parameters to reach optimal synthetic conditions. This concept echoes the operational paradigms of autonomous polymer laboratories, which leverage RL‐guided decision‐making to explore complex experimental landscapes in real time.

Furthermore, RL frameworks have been deployed for PK/PD optimization, dynamically modulating dosing regimens or nanoparticle size distributions to calibrate the equilibrium between therapeutic efficacy and systemic toxicity. The application of RL in pharmacometrics demonstrates its potential for the adaptive control of administration strategies, facilitating personalized and self‐optimizing dosing protocols. Prominent RL architectures, such as Deep Q‐Networks (DQN) and Policy Gradient algorithms (e.g., PPO, A3C), are trained using reward signals derived from experimental or simulated targets—such as percentage improvement in PK metrics, targeting indices, or release homogeneity. Recent literature underscores the expanding role of RL in robotics and autonomous experimentation, highlighting its integration into robotic control loops for adaptive materials design and nanoscale navigation [111, 112]. These methodologies establish a direct conduit between AI inference and empirical validation, transitioning RL from a mere simulation tool into a core driver of fully autonomous nanomedicine optimization.

In summary, RL provides a potent framework for adaptive, closed‐loop optimization in nanomedicine design. By synthesizing reward‐driven algorithms with automated experimental platforms, RL enables the real‐time discovery of optimal nanoparticle compositions and delivery strategies, marking a pivotal advancement toward the realization of autonomous nanopharmaceutical development.

### 3.4 PINNs and Hybrid Mechanistic–AI Models

To circumvent the inherent limitations regarding interpretability in purely data‐driven models, PINNs integrate established physical or pharmacokinetic governing equations directly into their loss functions. This architecture enforces network compliance with fundamental principles—such as mass conservation or diffusion kinetics—while retaining the inherent adaptive capacity of neural learning. Such hybrid modeling paradigms ensure scientific consistency, mitigate reliance on extensive datasets, and bolster predictive extrapolation across novel materials or physiological environments.

Within the context of PK/PD modeling, PINNs embed ordinary differential equations (ODEs) describing absorption, distribution, metabolism, and excretion (ADME) processes, facilitating the synchronous learning of model parameters and mechanistic interdependencies. Multiphysics pharmacokinetic models have successfully synthesized physiological transport equations with neural network architectures to simulate the inter‐tissue biodistribution of nanoparticles; these implementations demonstrate that physically constrained learning significantly elevates predictive accuracy and biological relevance [17, 113]. By encoding the governing ODEs of Physiologically Based Pharmacokinetic (PBPK) models into neural loss functions, PINNs maintain physiological interpretability, enabling robust generalization across diverse species and nanoparticle chemistries.

Regarding drug release kinetics, PINNs incorporate diffusion and degradation equations—such as the Higuchi, Korsmeyer‐Peppas, or erosion‐diffusion models—into their structural framework, yielding predictive models that strictly adhere to physical constraints while remaining adaptable to novel polymers or cross‐linking ratios. A mechanistic diffusion‐erosion model applied to surface‐erodible polymeric nanoparticles effectively captures both burst and sustained release phases, outperforming empirical regression approaches [114]. This hybrid formulation bridges the dichotomy between classical kinetic theory and data‐driven generalization, thereby enhancing transferability across distinct formulation classes.

Pertaining to targeting and biodistribution, hybrid mechanistic‐AI models fuse transport equations, such as vascular convection‐diffusion and receptor‐mediated transcytosis, with deep feature learning to simulate nanoparticle migration across endothelial barriers and within the tumor microenvironment. Recent multiscale PBPK frameworks illustrate how the integration of fluid‐transport physics with AI yields superior precision in predicting tissue‐specific accumulation compared to conventional compartmental models [7].

These hybrid architectures strike an optimal equilibrium between flexibility and interpretability—reducing data requirements, reinforcing biophysical reality, and facilitating cross‐domain transfer learning across diverse therapeutic modalities and administration routes. Although currently a conceptual roadmap rather than a fully deployed standard, integrating these models into advanced multi‐level AI frameworks promises to enable hierarchical learning: PINNs impose rigorous physical constraints at lower echelons, while deep networks perform abstract feature extraction at higher levels. Ultimately, this dual‐layered strategy provides an aspirational blueprint for constructing transparent, data‐efficient, and physiologically grounded nanomedicine models.

Navigating this diverse algorithmic landscape requires a systematic approach, conceptualized in the objective‐driven selection matrix in Figure 4. As illustrated, algorithmic selection must be anchored to the specific developmental stage: interpretable ML guides early screening, deep learning extracts complex structural topologies, PINNs enforce mechanistic fidelity (such as known physical mechanisms), and RL generates autonomous optimization trajectories.

Complementing this visual framework, Table 2 provides a highly granular, comparative summary of these models. As detailed in the table, the optimal model is heavily dictated by data constraints and specific objectives. For example, while neural networks (ANN, GPR, PINN) are highly recommended for capturing non‐linear drug release kinetics, their utility is fundamentally limited by temporal resolution. Conversely, while RL is unmatched for multi‐parameter closed‐loop tuning, it incurs a substantial computational cost. By aligning these algorithmic strengths with their corresponding limitations (Table 2), researchers can strategically construct the hybrid pipelines required for full‐scale in vivo translation.

**TABLE 2:** Model selection summary.

Objective	Recommended Models	Key Features / Advantages	Limitations
PK/PD prediction	RF, XGBoost, LSTM, PINNs [31, 115]	Handles structured and temporal data; interpretable	Sensitive to sparse sampling
Targeting and biodistribution	CNNs, GNNs, RF, Transformer [62, 116, 117]	Captures spatial patterns and feature interactions	Requires large, annotated datasets
Drug release kinetics	ANNs, GPR, PINNs [118, 119]	Learns nonlinear release dynamics; embeds diffusion physics	Limited temporal resolution
Multi‐parameter optimization	RL, Bayesian optimization [120, 121, 122]	Enables autonomous closed‐loop tuning	High computational cost

Aligning algorithmic strengths with specific data modalities and research objectives remains the critical prerequisite for the rational selection and training of AI models in nanotherapeutic design. Integrating the interpretability of classical machine learning, the representational power of deep learning, the optimization potential of reinforcement learning, and the mechanistic rigor of PINNs empowers researchers to construct robust, transparent, and adaptive AI systems that encompass the entire workflow—from data‐driven discovery to successful in vivo translation.

## 4 High‐Throughput Validation and Integration

High‐throughput experimental validation closes the iterative loop between AI‐driven predictions and empirical testing, facilitating the rigorous verification of computational hypotheses. Machine learning and reinforcement learning models actively inform experimental conditions, while emerging robotic systems and microfluidic technologies enable autonomous cycles of formulation, synthesis, and optimization.

AI‐generated design spaces frequently encompass hundreds or thousands of candidate formulations, rendering exhaustive manual validation unfeasible. Experimental verification of in vivo outcomes is therefore augmented by computational simulations that emulate complex biological processes. PBPK models and Agent‐Based Models (ABMs) serve as essential tools for validating AI‐predicted PK/PD profiles by reconstructing nanoparticle transport, biodistribution, and clearance within virtual biological systems. These models synthesize anatomical, physiological, and biochemical parameters—such as blood flow velocity, tissue permeability, and receptor density—to replicate spatiotemporal concentration profiles across diverse organs. The evolution of PBPK modeling permits increasingly precise predictions of nanoparticle biodistribution and systemic exposure across species, serving as a quantitative nexus between computational simulation and experimental observation [17, 123]. By integrating the mechanistic framework of PBPK models with AI‐generated formulation descriptors (e.g., size, zeta potential, lipid composition), researchers can quantify discrepancies between predicted and observed pharmacokinetic outcomes, thereby catalyzing the iterative refinement and validation of AI architectures [113].

DNA‐barcoding (bDNA) technology represents a transformative approach to high‐throughput in vivo testing, enabling the parallelized validation of large‐scale AI‐generated formulation libraries. Within this framework, individual nanoparticle formulations are tagged with unique oligonucleotide barcodes, allowing hundreds of candidates to be tracked concurrently within a single animal model. Systemic administration followed by tissue sequencing reveals the relative abundance of each barcode, providing multiplexed biodistribution data that directly validate AI‐predicted targeting efficiency [124]. Pioneered by the Dahlman lab, this methodology has revolutionized in vivo nanoparticle screening, driving the discovery of RNA delivery vehicles with distinct organ‐tropism; subsequent advancements have further expanded this platform to identify lipid nanoparticle formulations capable of selective RNA delivery to the bone marrow and other specific organs. Current bDNA‐based tools are indispensable for AI model validation, providing quantitative assessments of how nanoparticle composition, surface chemistry, and dimensions dictate organ‐level accumulation within a single experimental cycle. Complementary in vivo imaging modalities—including fluorescence imaging, PET, and MRI—permit spatiotemporal visualization of AI‐designed nanotherapeutics. Correlating imaging‐derived biodistribution maps with PBPK and AI predictions facilitates continuous parameter optimization. For example, time‐resolved PET imaging has been employed to validate the circulation half‐life and tumor‐accumulation kinetics of theranostic nanoparticles [125], whereas hybrid experimental‐modeling approaches utilize integrated PBPK frameworks to quantify the inhibitory effects of anti‐PEG antibodies on nanocarrier delivery [126]. Such imaging‐derived datasets enhance model interpretability by anchoring simulation‐based predictions to real‐time biodistribution and clearance dynamics across complex tissue architectures [127].

Predictive outcomes regarding drug release kinetics are increasingly validated via microdialysis and implantable biosensors, technologies that facilitate the real‐time quantification of localized drug concentrations under physiological conditions. Continuous monitoring of release behavior within specific tissues enables direct benchmarking of AI‐forecasted release curves against empirical pharmacodynamic datasets. PBPK modeling of fluorescent polymeric nanoparticles, for instance, has successfully replicated in vivo release kinetics observed through microdialysis assays [128]. Similarly, kinetic simulations of ceria nanoparticles demonstrate that particle degradation rates and surface charge exert a significant impact on observed in vivo release and clearance profiles [129].

These computational and experimental validation strategies furnish a biologically relevant benchmark for AI‐guided formulation design. Synthesizing PBPK simulations, bDNA multiplexed tracking, high‐resolution imaging, and real‐time release assays empowers researchers to iteratively refine AI architectures, ensuring predictive accuracy across PK/PD, targeting, and release domains while securing a closed‐loop transition from in silico results to in vivo therapeutic efficacy.

High‐throughput validation of in vitro data leverages automated technologies to fabricate and evaluate hundreds of AI‐predicted nanotherapeutic variants. Robotic systems equipped with liquid handling, precision mixing, and dynamic feedback control execute synthesis and optimization cycles with minimal human intervention. The “self‐driving” polymer laboratory pioneered by Tamasi et al. serves as a paradigm for this approach, integrating Bayesian optimization with automated synthesis and characterization to operationalize an iterative “Design‐Make‐Test‐Analyze”(DMTA) cycle [143, 144]. Comparable platforms, such as NanoMAP, synergize machine learning algorithms with robotic nanoprecipitation to facilitate the autonomous, multi‐objective optimization of polymer nanoparticle size, loading efficiency, and release rates [97, 145]. These infrastructures close the gap between model prediction and experimental reality, utilizing empirical feedback to augment AI architectures and inform subsequent in silico screenings [146, 147]. Microfluidic platforms have gained prominence by enabling precise control over reaction parameters—flow rates, temperatures, and solvent ratios—to achieve reproducible nanoparticle synthesis in micro‐scale volumes. Integration with real‐time sensors allows such systems to evaluate hundreds of formulation permutations under defined environmental constraints [137, 148]. Organ‐on‐a‐chip systems extend validation into physiologically relevant microenvironments, emulating vascular barriers, tumor tissues, or hepatic metabolism. Automated microplate‐based assays and optical sensor arrays enable the simultaneous measurement of release kinetics across dozens of formulations; correlating these data with AI predictions permits rapid verification of model veracity and the identification of deviations stemming from physicochemical instability or degradation. Robotic control minimizes inter‐experimental variance by ensuring uniform sampling intervals and environmental conditions, thereby providing high‐quality feedback for model retraining [149]. Consequently, in vitro assessments of uptake, permeability, and cytotoxicity prior to in vivo investigation offer quantitative validation for predictive PK/PD and targeting models [150, 151]. This frontier of automated validation is visualized in Figure 5. The central AI optimization engine relies on two distinct feedback loops: an automated in vitro screening platform (Figure 5A) that integrates robotic synthesis with tumor microenvironment‐on‐a‐chip models for multiparametric kinetic analysis, and a multiplexed in vivo platform (Figure 5B) that utilizes branched DNA (bDNA) barcoding to track dozens of lipid nanoparticle candidates concurrently, yielding high‐resolution biodistribution profiles. The apex of this integrated approach is exemplified by platforms such as the Lumi‐Lab (Figure 6). Driven by a pretrained molecular foundation model, the Lumi‐Lab hardware orchestrates full experimental loops—from high‐throughput synthesis via liquid handlers to real‐time readout and active learning refinement—allowing the system to autonomously propose, execute, and validate superior lipid candidates.

**FIGURE 5:**
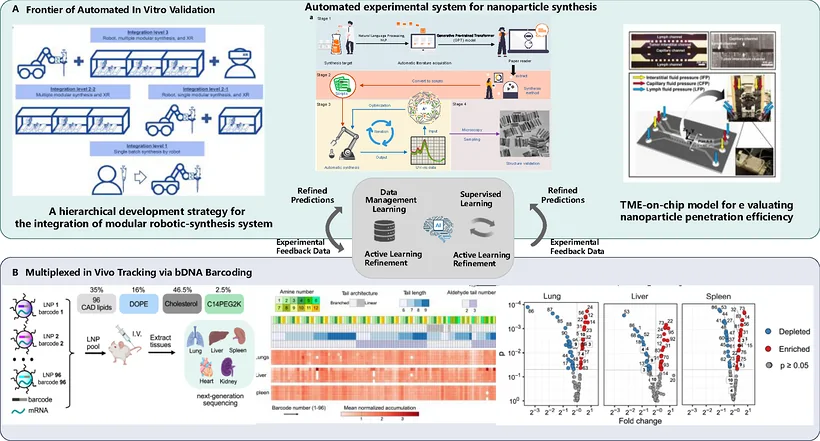
Schematic of the closed‐loop in vitro and in vivo validation framework for AI‐driven nanoparticle optimization. High‐throughput experimental testing enables dynamic retraining of the central AI engine toward predefined optimization targets. (A) Automated in vitro screening platform combining liquid‐handling robotics and multiwell formats. Tumor microenvironment‐on‐chip integration provides multiparametric analysis (spheroid models, physiochemical parameters, molecular kinetics), with assay data streaming directly into the central optimization loop [152, 153, 154]. (B) Multiplexed in vivo platform employing branched DNA barcoding for high‐throughput tracking of barcoded nanoparticles in murine models. Organ extraction coupled with next‐generation sequencing yields tissue‐specific biodistribution, PK, and Tox profiles, forming a continuous feedback loop for iterative model retraining and experimental refinement [132].

**FIGURE 6:**
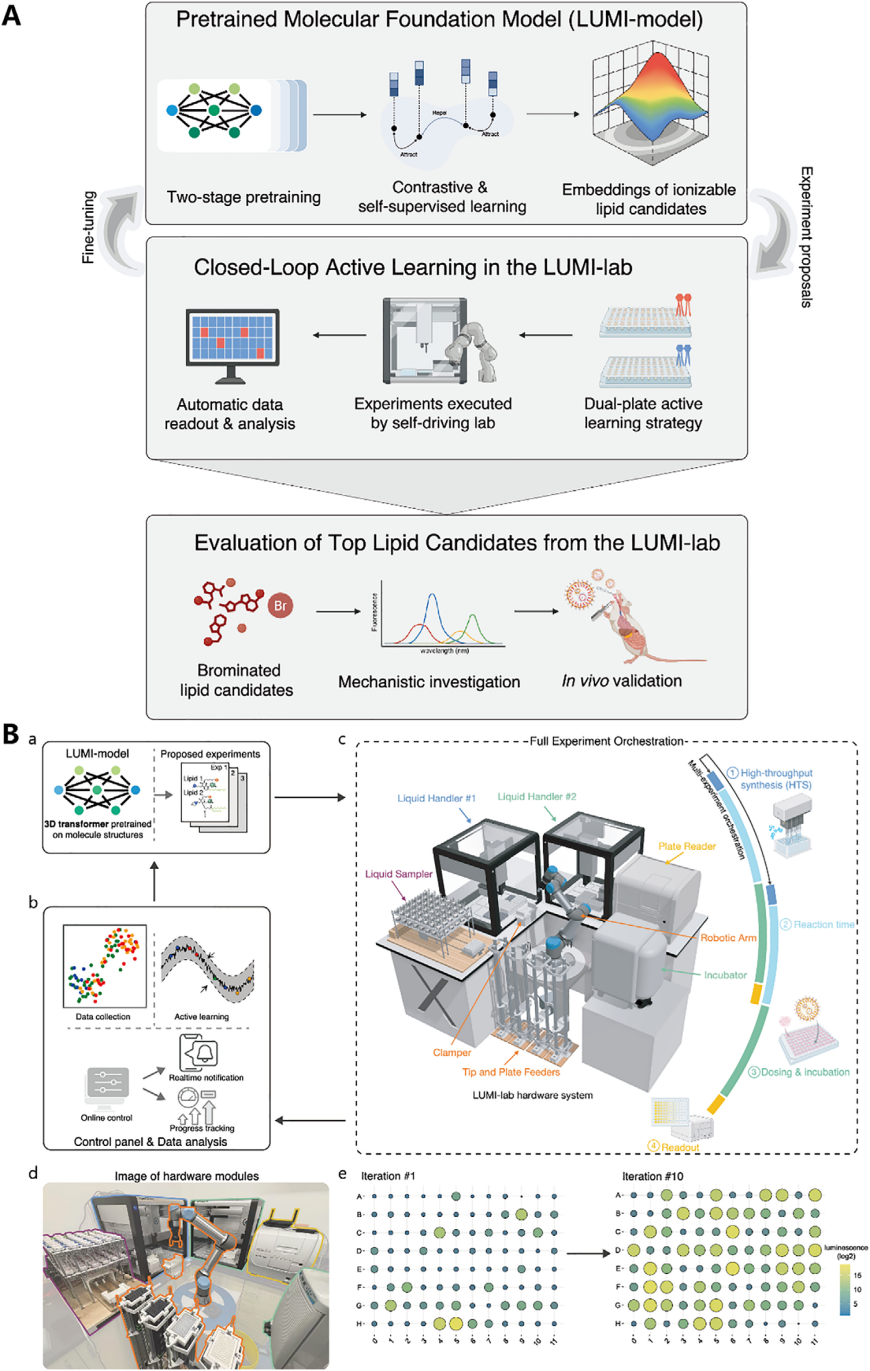
Lumi‐Lab: A transformer‐based foundation model‐driven self‐driving platform for LNP design—conceptual framework and operational workflow [136]. Reproduced with permission. Copyright 2026, Elsevier.

All derived in vivo and in vitro validation results necessitate integration into a unified data management system to facilitate continuous model enhancement. AI frameworks incorporating active learning dynamically select nascent experimental conditions based on uncertainty metrics, concentrating resources on data‐sparse regions with the lowest predictive confidence [155]. This integrated, high‐throughput validation ecosystem harmonizes AI forecasting, robotic synthesis, in vitro screening, in vivo confirmation, and model updating into a self‐reinforcing discovery and validation cycle. This modality fuses automation, computational modeling, and experimental validation into a continuous learning framework, significantly accelerating nanomedicine development and clinical translation. Automated in vitro workflows ensure scalability and reproducibility throughout the exploration of formulation landscapes. AI‐guided robotic laboratories now achieve autonomous synthesis, real‐time characterization, and active learning‐based decision‐making, generating multimodal, “AI‐ready” databases that consolidate synthesis, testing, and feedback information to refine nanotherapeutic optimization [109, 156]. Analogously, RL‐driven, autonomous flow‐based microreactors demonstrate the capability to expedite material optimization through closed‐loop AI experimentation [109]. While these validation ecosystems are diverse, their compatibility with closed‐loop AI optimization varies significantly, as systematically evaluated in Table 3. The table explicitly contrasts these technologies across throughput scale, core outputs, and AI integration readiness. For instance, while organ‐on‐a‐chip systems emulate physiologically relevant microenvironments to generate high‐quality feedback, their throughput remains at a medium‐high scale. In contrast, automated robotic systems evaluate hundreds of variants with “Very High”AI compatibility, seamlessly operationalizing the DMTA cycle. Therefore, as Table 3 highlights, while bDNA barcoding remains unparalleled for extracting multiplexed in vivo targeting profiles, robotic liquid‐handling platforms currently provide the most frictionless integration for continuous, autonomous model retraining.

**TABLE 3:** Comparative analysis of high‐throughput validation technologies: performance metrics and AI compatibility.

Validation Technology	Throughput Scale	Core Advantages & Key Outputs	Integration with AI & Closed‐Loop Compatibility
DNA‐barcoding (bDNA)	High (Tracks hundreds of candidates concurrently)	Enables parallelized in vivo testing of large‐scale formulation libraries. Yields multiplexed biodistribution data and quantitative assessments of organ‐level accumulation	High: Directly validates AI‐predicted targeting efficiency. The resulting multiplexed data enables continuous parameter optimization and iterative refinement of AI architectures [124, 130, 131, 132]
Automated Robotic Systems	High (Evaluates hundreds of AI‐predicted variants)	Executes synthesis and optimization cycles with liquid handling and precision mixing. Enables multi‐objective optimization of nanoparticle size, loading efficiency, and release rates.	Very High: Integrates with machine learning and Bayesian optimization to operationalize an autonomous “Design‐Make‐Test‐Analyze” (DMTA) cycle. Utilizes empirical feedback to continuously augment AI architectures [133, 134, 135, 136]
Microfluidic Platforms	High (Evaluates hundreds of formulation permutations)	Achieves reproducible nanoparticle synthesis in micro‐scale volumes. Provides precise control over reaction parameters such as flow rates, temperatures, and solvent ratios	High: Pairs with real‐time sensors to evaluate formulations under defined constraints. When driven by reinforcement learning (RL), it expedites material optimization through closed‐loop AI experimentation [137, 138, 139]
Organ‐on‐a‐chip Systems	Medium‐High (Simultaneous measurement across dozens of formulations)	Emulates physiologically relevant microenvironments (e.g., vascular barriers, tumor tissues). Provides quantitative assessments of uptake, permeability, and cytotoxicity prior to in vivo studies	Medium‐High: Correlating chip data with AI predictions permits rapid verification of model veracity. Minimizes inter‐experimental variance via robotic control, providing high‐quality feedback for model retraining [140, 141, 142]

Despite their clear advantages, integrating these high‐throughput validation platforms into a continuous DMTA workflow presents distinct limitations. Microfluidic systems, while offering precise control, frequently encounter scale‐up bottlenecks and channel clogging when translating from analytical to preparative scales. Organ‐on‐a‐chip platforms emulate local tissue interfaces but currently fall short of replicating the systemic immunobiological complexities and inter‐organ crosstalk essential for holistic PK/PD validation. Furthermore, although in vivo DNA‐barcoding enables high‐throughput multiplexing, it is constrained by high synthesis costs, potential barcoding‐induced alterations to nanoparticle physicochemical profiles, and the inherent risk of signal interference caused by premature payload leakage into the systemic circulation.

## 5 Discussion

AI‐integrated platforms have markedly accelerated nanotherapeutic development by enabling high‐throughput in silico screening, predictive optimization, and intelligent control of formulation parameters. Recent scholarly syntheses emphasize the capacity of deep learning and data‐driven modeling to streamline nanoparticle design, enhance translational predictability, and diminish reliance on heuristic trial‐and‐error experimentation [41]. DL architectures have demonstrated exceptional predictive capabilities in modeling nanoparticle biodistribution and protein corona evolution, thereby clarifying the ability of AI to resolve complex nano‐bio interactions and predict protein adsorption kinetics [40, 157]. Machine learning‐driven optimization has yielded substantial gains in the delivery efficiency of lipid nanoparticles (LNPs). For instance, Wang et al. utilized AI‐guided in silico screening to identify novel ionizable lipids with superior mRNA delivery performance [158], while Pei et al. employed RF models to refine LNP composition and architecture, achieving a fourfold increase in targeted delivery efficiency [159]. Similarly, Xu et al. demonstrated the capability of the AGILE platform to accelerate the screening of over 10 000 lipid candidates via deep learning and combinatorial chemistry [83]. Reinforcement learning frameworks and closed‐loop robotic systems have realized the autonomous optimization of nanomedicines, synthesizing AI‐based forecasting with robotic synthesis and real‐time validation [145]. Within microfluidic environments, reinforcement learning facilitates adaptive process control, thereby enhancing formulation yield and reproducibility [121]. These findings underscore the potential of AI to bridge the gap between computational prediction, empirical validation, and clinical translation. Integrating deep learning, machine learning, and reinforcement learning into robotic and microfluidic platforms establishes a continuous “Design‐Make‐Test‐Analyze” (DMTA) cycle, which rapidly converges toward optimal nanotherapeutic performance while curtailing developmental timelines and empirical costs. To encapsulate this paradigm shift, Figure 7 presents a comprehensive roadmap from in silico conceptualization to clinical adoption. The critical feature of this trajectory is not its linearity, but the overarching macro‐loop: real‐world clinical feedback and patient‐specific data must continuously flow back into the foundational databases, ensuring the core AI engines evolve and self‐correct with each translational cycle.

**FIGURE 7:**
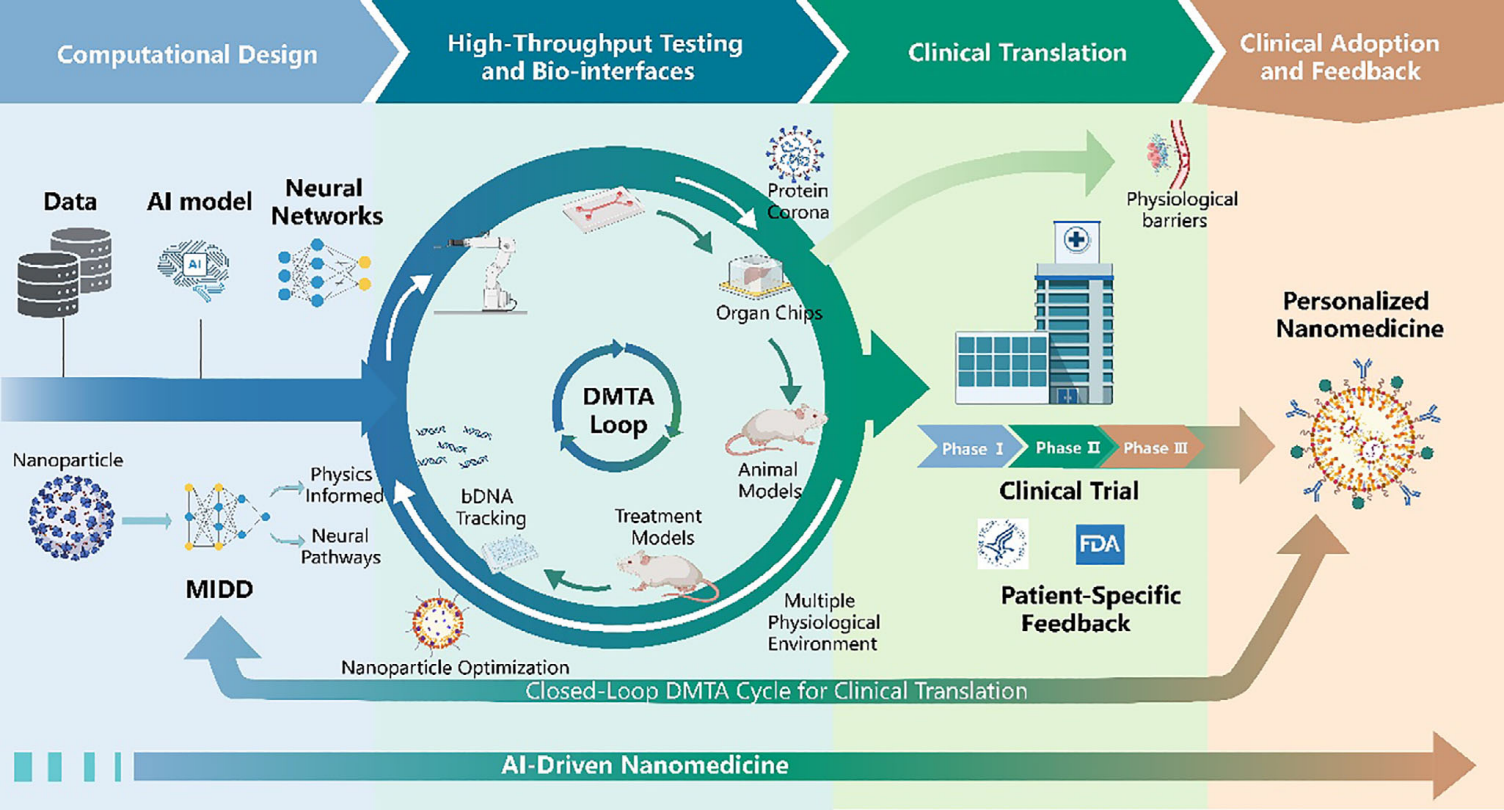
A Roadmap for the AI‐Driven Discovery Ecosystem. This schematic illustrates the necessary integration of computational design, high‐throughput automated synthesis, and multiplexed biological testing to form a closed‐loop “Design‐Make‐Test‐Analyze” (DMTA) cycle, accelerating the translation of reproducible and mechanistically interpretable nanomedicine candidates into the clinic.

Despite these advancements, persistent challenges continue to impede AI‐driven nanomedicine development. Data quality and heterogeneity represent critical bottlenecks, as the lack of standardization and cross‐laboratory compatibility limits model generalizability and reproducibility. Establishing FAIR‐compliant (Findable, Accessible, Interoperable, and Reusable) data repositories is imperative to catalyze progress in this domain [160]. Model interpretability remains a substantive hurdle for regulatory adoption, as deep learning architectures often function as “black boxes,” obfuscating the mechanistic underpinnings of predictive outcomes. Synthesizing mechanistic modeling—such as PBPK models or PINNs—with data‐driven AI serves to enhance the transparency and credibility of these platforms [161, 162]. Validation frameworks must concurrently evolve; closed‐loop AI‐robotics systems and hybrid virtual‐experimental models bolster reproducibility while reducing reliance on animal studies, in alignment with the 3R principles. Regulatory frameworks, such as the FDA's MIDD and guidelines for AI/ML‐based medical devices, have begun acknowledging AI‐derived evidence as a legitimate supplementary validation modality [163].

Overcoming the translational chasm between preclinical animal models and human physiology remains paramount for the clinical viability of AI‐designed nanomedicines. Bridging this gap necessitates advanced transfer learning strategies, wherein neural network weights optimized on extensive in vivo murine datasets are systematically fine‐tuned using sparse, highly curated clinical data [164]. Concurrently, the integration of cross‐species Physiologically Based Pharmacokinetic (PBPK) models allows researchers to recalibrate AI predictions according to human‐specific anatomical and metabolic parameters. Equally critical is overcoming the “black‐box” paradigm that impedes regulatory acceptance. Utilizing Explainable AI (XAI) frameworks—such as SHAP and LIME [165]—in conjunction with Model‐Informed Drug Development (MIDD) protocols empowers researchers to explicitly map AI‐derived formulation permutations to underlying physicochemical and biological drivers [63]. This transparency not only demystifies the predictive rationale but also aligns the AI‐driven nanotherapeutic development process with the rigorous mechanistic expectations of regulatory agencies [163].

Looking forward, the future of nanomedicine relies on transcending isolated computational tools to build fully integrated, closed‐loop discovery ecosystems. The convergence of AI, automated robotic synthesis, and multiplexed biological testing will soon enable the autonomous generation of nanotherapeutics. By demanding that these systems remain anchored to mechanistically interpretable physical and biological constraints, the field can finally overcome the historical trial‐and‐error bottleneck, paving a reproducible and highly efficient path toward clinically translated nanomedicines.

## Author Contributions

**Meichen Dong**: conceptualization, investigation, visualization, writing – original draft. **Qin Luo**: writing – review and editing. **Xinyi Ding**: writing – review and editing. **Chaoqi Lu**: writing – review and editing. **Ru Zhang**: writing – review and editing. **Qingqing Tan**: writing – review and editing. **Zhiqing Pang**: writing – review and editing. **Defang Ouyang**: writing – review and editing. **Dongsheng Li**: funding acquisition, writing – review and editing. **Jiaxuan Xia**: funding acquisition, writing – review and editing. **Jianxin Wang**: funding acquisition, writing – review and editing.

## Funding

The work was supported by Shanghai‐Yunnan Science and Technology Collaborative Innovation Project (No. 26HD0700200 and 202603AD140004), National Natural Science Foundation of China (No. 82374296), Shanghai “Science and Technology Innovation Action Plan” Biomedical Innovation Development Project (No. 24S11903000), the Postdoctoral Fellowship Program of China Postdoctoral Science Foundation (Grant No. GZC20252374), and the China Postdoctoral Science Foundation General Program (Grant No. 2024M760568).

## Conflicts of Interest

The authors declare no conflicts of interest.

## Data Availability

The data that support the findings of this study are available from the corresponding author upon reasonable request.
